# Exploring the existence of a stayer population with mover–stayer counting process models: application to joint damage in psoriatic arthritis

**DOI:** 10.1111/rssc.12187

**Published:** 2016-10-19

**Authors:** Sean Yiu, Vernon T. Farewell, Brian D. M. Tom

**Affiliations:** ^1^ Medical Research Council Biostatistics Unit Cambridge UK

**Keywords:** Intermittent observations, Longitudinal count data, Mover–stayer model, Poisson process, Psoriatic arthritis, Random effects

## Abstract

Many psoriatic arthritis patients do not progress to permanent joint damage in any of the 28 hand joints, even under prolonged follow‐up. This has led several researchers to fit models that estimate the proportion of stayers (those who do not have the propensity to experience the event of interest) and to characterize the rate of developing damaged joints in the movers (those who have the propensity to experience the event of interest). However, when fitted to the same data, the paper demonstrates that the choice of model for the movers can lead to widely varying conclusions on a stayer population, thus implying that, if interest lies in a stayer population, a single analysis should not generally be adopted. The aim of the paper is to provide greater understanding regarding estimation of a stayer population by comparing the inferences, performance and features of multiple fitted models to real and simulated data sets. The models for the movers are based on Poisson processes with patient level random effects and/or dynamic covariates, which are used to induce within‐patient correlation, and observation level random effects are used to account for time varying unobserved heterogeneity. The gamma, inverse Gaussian and compound Poisson distributions are considered for the random effects.

## 1 Introduction

In psoriatic arthritis, several researchers (Aguirre‐Hernández and Farewell (2004), Solis‐Trapala and Farewell (2005) and recently O’Keeffe *et al*. (2012)) have considered the existence of a stayer population (those who do not have the propensity to experience the event of interest) with regard to clinical joint damage, after having discussed the clinical plausibility of such a population with Professor Dafna Gladman, who has established the largest and most comprehensively studied cohort of psoriatic arthritis patients in the world. If a subpopulation of stayers exists, the identification of characteristics that are associated with being a stayer would provide further understanding of the disease, as would accounting for such a population when characterizing the rate of developing damaged joints in the movers (those who have the propensity to experience the event of interest). Since these studies, there has been even more empirical evidence to suggest that a stayer population may exist; many patients have remained damage free even after further follow‐up. Thus it now seems particularly appropriate to provide a more comprehensive investigation into the existence of a stayer population.

Because the outcome is the occurrence of permanently damaged joints over time, mover–stayer counting process models will be considered as a useful framework for analysis. In the literature, the models for the movers are typically constructed based on Poisson processes with patient or observation level random effects; these act multiplicatively on the intensity for greater flexibility. In addition to these commonly proposed models, models with both patient and observation level random effects and where the random effects are of a compound Poisson (CP) form will also be considered. This paper demonstrates that, when fitted to the same data (real psoriatic arthritis data and simulated data), these models can provide widely varying conclusions on the existence and proportion of a stayer population, thus demonstrating that, as is commonly done in the literature, a single analysis of a stayer population should not generally be adopted uncritically. In light of these results and given the prevalence of longitudinal count data with excess 0s, this paper aims to provide greater understanding of mover–stayer counting process models, particularly relating to estimation of the stayer proportion, by comparing the inferences, performance and model features of many models fitted to real and simulated data sets. We note that these results are analogous to those obtained on cure rate models (Boag, 1949; Farewell, 1977, 1982, 1986; Gordan, 1990; Ghitany *et al*., 1994) where it has been shown that the estimate of the cured fraction can be quite sensitive to the choice of the survival distribution (Yu *et al*., 2004).

Aguirre‐Hernández and Farewell (2004) and Solis‐Trapala and Farewell (2005) have previously used mover–stayer counting process models to investigate the existence of a stayer population that does not develop damaged joints. They considered models for the movers based on introducing independent gamma random effects into Poisson process (Kingman, 1992) models at the observation and patient level respectively. By comparing inferences with their respective non‐mover–stayer model, neither of these investigations found any convincing evidence for a stayer population. The former analysis was performed at the total joint level, using the overall total joint count, whereas the latter was performed at the total joint level, using only the counts for the hand joints. O’Keeffe *et al*. (2012) took a different approach based on the use of mover–stayer multistate models at the individual hand joint level. They considered several patient level random‐effects distributions, the gamma, inverse Gaussian (IG) and CP distribution, and demonstrated large discrepancies in the estimates for the probability of being a stayer (in the hands) across the different random‐effects distributions. In particular, the use of patient level gamma random effects in the model for the movers produced little evidence for a stayer population, whereas more convincing evidence was found when patient level IG and CP random effects were used instead. Although the analyses performed by Aguirre‐Hernández and Farewell (2004), Solis‐Trapala and Farewell (2005) and O’Keefe *et al*. (2012) were on different versions of the same data set and the modelling approach of O’Keeffe *et al*. (2012) was different (multistate models as opposed to counting process models), the discrepancies across the various random‐effects distributions may suggest that the results of Aguirre‐Hernández and Farewell (2004) and Solis‐Trapala and Farewell (2005) were linked to the choice of gamma random effects and that, if a different random‐effects distribution was chosen, vastly different results and conclusions may have occurred. This paper builds on this important issue (different random‐effects distributions providing vastly different conclusions on a stayer population) observed by O’Keeffe *et al*. (2012) by also considering other random‐effects structures, so that the results are more general or relevant to the count data setting, and additionally considering model performance and simulation studies to provide greater understanding.

The next section introduces the psoriatic arthritis data on which this analysis is based.

## 2 Psoriatic arthritis and the data

Psoriatic arthritis is an inflammatory arthritis associated with psoriasis. A basic measure of disease progression in psoriatic arthritis is the number of permanently damaged joints. Apart from the damage process being permanent (once a joint becomes damaged it will remain so), damaged joints can ultimately lead to disability and a reduced quality of life (Husted *et al*., 2001; Kane *et al*., 2003). It is therefore important to prevent or slow the damage process where possible. The main strategy employed by clinicians is to reduce activity in the joints (swelling and/or pain in the joints) as this is widely believed to result in or to cause joint damage (O’Keeffe *et al*., 2011). This paper focuses on the 28 joints in the hands (14 joints in each hand), which can result in severe disability if damaged. Analyses will be based on data from the University of Toronto psoriatic arthritis clinic which, since its inception in 1978 until the start of 2013, has followed over 1000 patients with clinic visits scheduled approximately 6–12 months apart. At each clinic visit, a physical examination, routine blood and urine tests and a rheumatological assessment which includes a count of active and damaged joints are performed.

To produce a more homogeneous set of patients, we considered the 757 patients who entered the clinic with no damaged hand joints and had more than a single clinic visit. Of these patients 422 (55.75%) were male and 335 were female (44.25%). At clinic entry, the mean age was 42.19 years, with a standard deviation of 12.48 years. The mean and median number of clinic visits per patient were 11.27 and 7, and this ranged from 2 to 57. The mean age at onset of arthritis was 36.76 years, with a standard deviation of 13 years. The mean follow‐up time was 9.46 years, with an interquartile range of 11.15 years. The mean and median intervisit times were 0.84 and 0.54 years, with a standard deviation of 1.19 years.

While in the clinic, a large percentage, 72% (524 patients), of these patients remained damage free in the hand joints. Of the patients (233 patients) who developed damaged joints, the mean rate of gaining damage was 0.53 joints per year (total number of damaged joints at the last clinic visit or follow‐up time in the clinic).

For this subset of the data, we consider models to estimate the proportion of stayers and to characterize the rate of developing damage in the movers. Although the development of damaged hand joints is not formally a recurrent events process (because there is a finite number of hand joints), the models for the movers are based on Poisson processes, as an approximation, with various random‐effects structures and distributional assumptions. Heuristically, recurrent events methodology was considered as a reasonable approximation because there are few occasions where a large number of damaged hand joints have been observed, and therefore these observations will contribute less in the analysis. More formal justifications are provided in Sections 3.4 and 5.2.

## 3 Patient level random‐effects models and observation level random‐effects models

### 3.1 Patient level random‐effects models

Let $D_{ij}=N_{ij+1}-N_{ij}$ be the number of damaged joints that patient *i* has developed between the *j*th and (*j*+1)th clinic visit. A natural first choice would be to assume that $D_{ij}$ is Poisson distributed with mean

$$u_{i}\Lambda_{ij}=u_{i}\left(t_{ij+1}-t_{ij}\right)\lambda_{0}\text{exp}\left(\beta^{\prime }z_{ij}\right)$$

where $u_{i}$ is a realization of the patient level random effect $U_{i}$ which induces correlation between the observations of a patient, ${\left\{t_{ij}\right\}}_{j=0}^{m_{i}}$ are the times at which the clinic visits occurred, $\lambda_{0}$ is a constant baseline intensity and ***β*** and $z_{ij}$ are column vectors of regression coefficients and covariates evaluated at the *j*th clinic visit respectively. To account for a subpopulation of stayers, the distribution of $U_{i}$ is taken to have a mixture distribution. Specifically the mover–stayer random effects densities for $U_{i}$ are of the form

$$g_{M-S}\left(u_{i}\right)=\left\{\begin{array}{cc} \pi_{i}, & \text{if}\;u_{i}=0,\\ f(u_{i}), & \text{if}\;u_{i}>0, \end{array}\right.$$

where $\pi_{i}$ is the probability that patient *i* is a stayer and $f(u_{i})$ is a truncated random‐effect density which integrates to $1-\pi_{i}$ when patient *i* is a mover. The corresponding marginal likelihood contribution from patient *i* is then

$${\left\{\;\int_{0}^{\infty }\prod_{j=0}^{m_{i}-1}\frac{{\left(u_{i}\Lambda_{ij}\right)}^{d_{ij}}\text{exp}\left(-u_{i}\Lambda_{ij}\right)}{d_{ij}!}f\left(u_{i}\right)du_{i}\right\}}^{c_{i*}}{\left\{\pi_{i}+\int_{0}^{\infty }\prod_{j=0}^{m_{i}-1}\text{exp}\left(-u_{i}\Lambda_{ij}\right)f\left(u_{i}\right)du_{i}\right\}}^{1-c_{i*}}$$

where $c_{i*}=0$ if patient *i* remained damage free while in the clinic and $c_{i*}=1$ otherwise. The likelihood is then constructed by taking the product of all likelihood contributions from patients in the data set. Models corresponding to a likelihood of this form will be referred to as Poisson mover–stayer models and further qualification, when needed, will be through the addition of the type of random‐effects distribution used.

Subsequently, the three different random‐effects distributions that were used by O’Keeffe *et al*. (2012) will be considered. The first two random‐effects distributions are of the form

$$g_{M-S}\left(u_{i}\right)=\left\{\begin{array}{cc} \pi_{i}, & \text{if}\;u_{i}=0,\\ \left(1-\pi_{i}\right)g\left(u_{i}\right), & \text{if}\;u_{i}>0, \end{array}\right.$$

where $g(u_{i})$ has either a gamma density with rate and shape parameter 1/*θ* or an IG density with mean 1 and shape parameter *ψ*, i.e.

$$g\left(u_{i}|\theta \right)=\frac{{\left(1/\theta \right)}^{1/\theta }}{\Gamma \left(1/\theta \right)}u_{i}^{1/\theta -1}\text{exp}\;\;\left(\;\;-\frac{u_{i}}{\theta }\right)$$

or

$$g\left(u_{i}|\psi \right)={\left(\frac{\psi }{2\pi u_{i}^{3}}\right)}^{1/2}\text{exp}\;\left\{\;\;-\frac{\psi {\left(u_{i}-1\right)}^{2}}{2u_{i}}\right\}.$$

These two distributions will be referred to as the mover–stayer gamma and mover–stayer IG models respectively. The third mover–stayer random‐effects density is a CP density of the form

$$U_{i}=\sum_{j=1}^{N_{i}}X_{\;\;j},$$

where $N_{i}$ is a Poisson random variable with rate parameter $\rho_{i}$ and $X_{j}$ ($j=1,\ldots ,N_{i}$) are independently and identically distributed gamma random variables with shape and rate parameters 1 and *ν* respectively. The density is given by

$$g_{M-S}\left(u_{i}\right)=\left\{\begin{array}{cc} \text{exp}(-\rho_{i}), & \text{if}\;u_{i}=0,\\ \text{exp}\left(-\rho_{i}-\nu u_{i}\right)\surd \left(\nu \rho_{i}/u_{i}\right)I_{1}\left\{2\surd \left(\nu \rho_{i}u_{i}\right)\right\}, & \text{if}\;u_{i}>0, \end{array}\right.$$

where $I_{1}\left(h\right)$ is a modified Bessel function of the first kind, i.e.

$$I_{1}\left(h\right)=\sum_{k=0}^{\infty }\frac{1}{k\Gamma (k+2)}{\left(\frac{h}{2}\right)}^{2k+1}.$$

Aalen (1992) and Moger (2004, 2005) have provided applications of the CP distribution to survival studies.

Another commonly used random‐effects distribution for the positive part is the log‐normal distribution. It can, however, be shown that this distribution is very similar to an IG distribution and hence we do not consider its use here as it affords less tractability. See Chhikara and Folks (1977) for more details.

It is also worth noting that there is a more general distribution which has the gamma, IG and CP distributions nested in its family. The power variance family function is a three‐parameter family that was introduced by Hougaard (1986) and extended in Hougaard (2000). Its density and Laplace transform are

$$g\left(u|m,\nu ,\rho \right)=\text{exp}\left(-\rho -\nu u\right)\frac{1}{u}\sum_{j=1}^{\infty }\frac{\rho^{j}{\left(\nu u\right)}^{mj}}{\Gamma (mj)j!}$$

and

$$L\left(s,m,\nu ,\rho \right)=\text{exp}\;\;\left[-\rho \left\{1-{\left(\frac{\nu }{\nu +s}\right)}^{m}\right\}\right]$$

where *m*>−1, *ν*>0 and *mρ*>0. When *ρ*→∞ and *m*→0 at a rate such that *ρm*→*η*, the gamma distribution results. For the cases where $m=-\frac{1}{2}$ and *m*>0, the IG and CP distributions arise. In certain more tractable situations, i.e. when its Laplace transform is only required, the parameter *m* can be estimated directly, thus providing information on the existence (*m*>0) or non‐existence (*m*⩽0) of a stayer population. Note that the special case of *m*=1 corresponds to the CP distribution that is used in this paper.

### 3.2 Observation level random‐effects models

The Poisson mover–stayer models are derived by conditioning on a single random effect for each patient to give conditionally independent contributions from a patient's data. This induces a correlation structure between the observations of a patient. This patient‐specific random effect $U_{i}$ might be thought of as accounting for missing time invariant variables that generate heterogeneity across patients. It is, however, plausible that such unobserved variables are not constant with time, especially when the follow‐up periods are as long as in the psoriatic arthritis data set. For example a patient's prescribed medication, which is administrated at each clinic visit, can dramatically change with time. Another commonly adopted approach would be to retain an individual‐specific probability of being at no risk of damage, $\pi_{i}$, but to condition each observation from patients at risk on a different random effect $U_{ij}$ drawn from an appropriate distribution. This would allow the unobserved heterogeneity across patients to vary with time also.

If this approach is adopted, some adjustment for the correlation between the observations on the same patient, if they are at risk of damage, can be induced by inclusion of the observed dynamic variable $N_{ij}$, which specifies the attained number of damaged joints at the *j*th visit. Thus the assumption becomes one of independence between incremental damage observations from the same patient given this observed dynamic variable and the patient being in the at‐risk group.

More formally, $D_{ij}$ is assumed Poisson distributed with mean

$$c_{i}u_{ij}\Lambda_{ij}=c_{i}u_{ij}\left(t_{ij+1}-t_{ij}\right)\lambda_{0}\text{exp}\left(\beta^{\prime }z_{ij}\right),$$

where

$$c_{i}=\left\{\begin{array}{cc} 1, & \text{with probability}\;1-\pi_{i},\\ 0, & \text{with probability}\;\pi_{i}, \end{array}\right.$$

indicates whether the *i*th patient is a mover ($c_{i}=1$) or stayer ($c_{i}=0$). The corresponding marginal likelihood contribution from patient *i* is then

$$\begin{array}{cc} & {\left\{\left(1-\pi_{i}\right)\prod_{j=0}^{m_{i}-1}\int_{0}^{\infty }\frac{{\left(u_{ij}\Lambda_{ij}\right)}^{d_{ij}}\text{exp}\left(-u_{ij}\Lambda_{ij}\right)}{d_{ij}!}g\left(u_{ij}\right)du_{ij}\right\}}^{c_{i*}}\\ & \times {\left\{\pi_{i}+\left(1-\pi_{i}\right)\prod_{j=0}^{m_{i}-1}\int_{0}^{\infty }\text{exp}\left(-u_{ij}\Lambda_{ij}\right)g\left(u_{ij}\right)du_{ij}\right\}}^{1-c_{i*}}. \end{array}$$

The dynamic variable $N_{ij}$ enters through being an element in $z_{ij}$. Note that the integration over the random effects that is required in the marginal likelihood contribution of patient *i* is performed at the observation structural level and therefore $m_{i}$ integrations are required. This contrasts with the marginal likelihood for the Poisson mover–stayer models which contained a patient level random effect for which a single integration was performed over the contributions from all observations on a single patient. Although these models are again of the mover–stayer structure, they will be referred to as zero‐inflated models for consistency with the literature. Note that the 0‐inflation is at the patient and not observation level (i.e. $c_{i}$ and not $c_{ij}$ is considered) and therefore a patient can only be either a stayer or a mover throughout their follow‐up. Further qualification of the zero‐inflated models will again be through the marginal models that are used for patients in the at‐risk category.

### 3.3 Model fitting

For the Poisson mover–stayer models, the gamma and IG distributional parts of the mover–stayer random‐effects distributions were parameterized to have unit means to avoid identifiability problems with the baseline intensity. An alternative, but mathematically equivalent, approach to avoid non‐identifiability was taken for the CP random‐effects distribution which has an expectation of $\rho_{i}/\nu ;\;\rho_{i}$ and *ν* were allowed to vary freely with $\lambda_{0}$ constrained to 1.

The three Poisson mover–stayer models corresponding to these three random‐effects distributions are referred to as the Poisson mover–stayer gamma, Poisson mover–stayer IG and the Poisson mover–stayer CP models. The zero‐inflated models were examined when $g(u_{ij})$ was taken separately to be gamma and IG distributions, resulting in the zero‐inflated negative binomial (ZINB) and zero‐inflated Poisson IG (ZIPIG) models as they are commonly known in the literature. The same parameterizations for the gamma and IG distributions from Section 3.1 were used, although their rate and shape parameters are denoted by $\theta_{ij}^{\mathrm{nb}}$ and $\psi_{ij}^{\mathrm{pig}}$ respectively. Here the subscripts on the dispersion parameters indicate the possible dependence on explanatory variables from the *i*th patient at $t_{ij}$. The CP distribution was not considered as an observation level random‐effects distribution because a mass at zero exists in this distribution and hence would represent the unrealistic scenario that a patient can be a stayer or mover at one point in time and a mover or stayer at another. This is unlike the case when patient level random effects are considered, where a single random effect specifies whether a patient is a mover or stayer.

Parameter estimation for these and all subsequent models in this paper was achieved by maximizing the log‐likelihood function by using the ‘Broyden–Fletcher–Goldfarb–Shanno’ optimization technique (Broyden, 1970) in the statistical package R (R Core Team, 2015). The analytic solutions to the integrals in the likelihood for the random‐effects distributions that are considered in this section can be found in the on‐line appendix A. The confidence intervals for all parameters and quantities reported are 95% Wald intervals obtained from evaluating and then inverting the observed information matrix at the maximum likelihood estimates.

The number of currently active (swollen and/or painful) joints, permanently attained damaged joints, age at onset of arthritis (in years) and duration of arthritis (in years) were considered as explanatory variables for the mean functions, and intercept‐only models were considered for the dispersion parameters, i.e. $\theta_{ij}^{\mathrm{nb}}=\theta^{\mathrm{nb}}$ and $\psi_{ij}^{\mathrm{pig}}=\psi^{\mathrm{pig}}$.

All models were fitted with $\pi_{i}=\pi$ so that the existence of a stayer population could be more simply investigated, specifically, through testing the null hypothesis $H_{0}:\pi =0$ for the Poisson mover–stayer gamma, Poisson mover–stayer IG and the zero‐inflated models. Under the null hypothesis, the asymptotic distribution of the likelihood ratio test for these models (against their non‐mover–stayer counterpart) is a 50:50 mixture of a point mass at zero and a $\chi_{1}^{2}$‐distribution (Self and Liang, 1987). A test of $H_{0}:\pi =0$ for the Poisson mover–stayer CP model is equivalent to testing $H_{0}:\text{exp}\left(-\rho \right)=0$ (or $H_{0}:\rho =\infty$). However, under this null hypothesis, the parameter *ν* becomes irrelevant (see the on‐line appendix B for details), which therefore results in the asymptotic distribution of the likelihood ratio statistic being intractable. For this model, consideration will initially be given to the 95% Wald interval of $\hat{\pi }$ to reason about the existence of a stayer population. It is also worth noting that models where $\pi_{i}$ depends on explanatory variables can be developed. However, this would make sense only if reasonable evidence exists for a subpopulation of stayers.

### 3.4 Results

Table 1 shows the results from the Poisson mover–stayer (patient level random‐effects) and zero‐inflated (observation level random‐effects) models fitted to the Toronto psoriatic arthritis data described in Section 2. Within the class of Poisson mover–stayer and zero‐inflated models, the regression coefficient estimates are quite similar; most estimates lie in the corresponding 95% Wald interval of the other models. The current number of active joints, duration of arthritis and age at onset of arthritis demonstrate significant positive associations with damage progression in the Poisson mover–stayer models, whereas the current number of active joints is seen to have a significant positive association in the zero‐inflated models. The Poisson mover–stayer models suggest that the attained number of damaged joints has a negative association with damage progression whereas the zero‐inflated models suggest a positive association. After accounting for correlation through the multiplicative patient level random effect, it is natural to postulate that the negative association is indicative of there being fewer joints having the propensity to become damaged. This would suggest that different results may have been obtained if the number of joints that are at risk of damage was accounted for in the model (beyond inclusion of this dynamic covariate). For this reason, truncated Poisson models and multistate models at the individual joint level were fitted; these models directly account for the number of joints that are at risk of damage at each time point. Reassuringly, similar results were produced including the significant negative or positive association of the attained number of damaged joints when patient or observation level random effects were included. As mentioned, the attained number of damaged joints was primarily introduced to provide information about the history of a patient due to true contagion and is therefore introducing correlation between the patient observations. As the patient level random effect is also designed to reflect partly this type of correlation (in addition to capturing time invariant unobserved heterogeneity), the effect of this dynamic covariate will probably be confounded with the random effects. For a detailed discussion on the connection between frailty models and models with dynamic covariates see Aalen *et al*. (2008).

**Table 1:** Parameter estimates and corresponding 95% Wald intervals of associations with damaged joint counts and stayer probability estimates obtained from fitting the Poisson mover–stayer (patient level random‐effects models) and zero‐inflated models (observation level random‐effects models) to 757 psoriatic arthritis patients

	*Results for the following models:*				
	*Poisson mover–*	*Poisson mover–*	*Poisson mover–*	*ZINB*	*ZIPIG*
	*stayer gamma*	*stayer IG*	*stayer CP*		
Attained number of damaged joints	−0.11	−0.12	−0.082	0.059	0.057
	(−0.13,−0.095)	(−0.14,−0.1)	(−0.097,−0.066)	(0.033, 0.084)	(0.033, 0.081)
Current number of active joints	0.061	0.06	0.064	0.095	0.097
	(0.047, 0.075)	(0.046, 0.074)	(0.051, 0.078)	(0.068, 0.12)	(0.072, 0.12)
Arthritis duration	0.07	0.074	0.045	0.0063	−0.0022
	(0.056, 0.084)	(0.059, 0.088)	(0.033, 0.056)	(−0.0063,0.019)	(−0.016,0.011)
Age at onset of arthritis	0.021	0.02	0.014	0.0078	−0.00045
	(0.0051, 0.037)	(0.0015, 0.038)	(0.0031, 0.024)	(−0.0031,0.019)	(−0.012,0.011)
$\lambda_{0}$	0.034	0.052	1	0.1	0.16
	(0.018, 0.065)	(0.024, 0.12)		(0.061, 0.17)	(0.093, 0.27)
*θ*	6.19				
	(5.11, 7.5)				
*ψ*		0.11			
		(0.066, 0.19)			
*ν*			12		
			(6.68, 17.32)		
*ρ*			0.58		
			(0.49, 0.67)		
$\theta^{\mathrm{nb}}$				9.78	
				(8.1, 11.45)	
$\psi^{\mathrm{pig}}$					0.062
					(0.048, 0.081)
*π*	9.1$\times 10^{-4}$	0.32	0.56	0.43	0.44
	(0, 1)	(0.2, 0.47)	(0.51, 0.61)	(0.36, 0.5)	(0.37, 0.51)
Log‐likelihood	−2689.03	−2676.34	−2741.55	−2279.36	−2273.27

**Figure 1:**
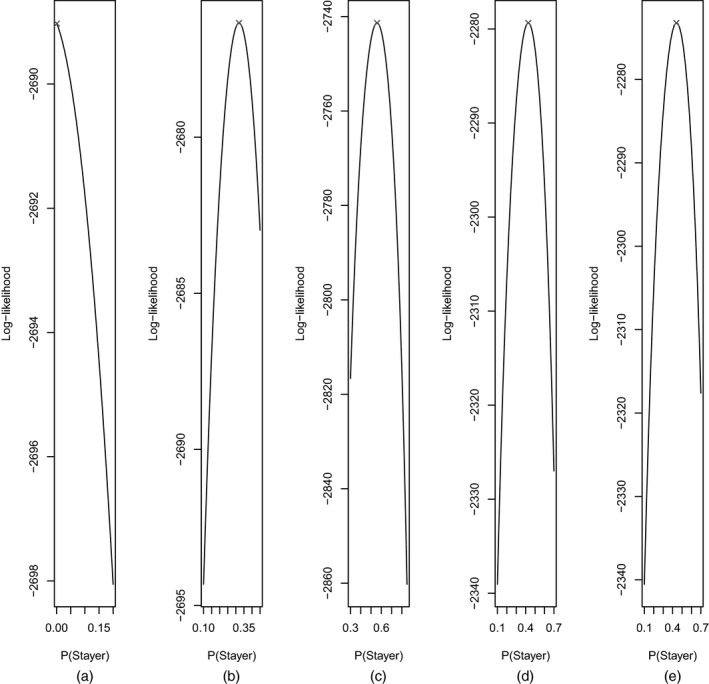
Plots of the profile log‐likelihoods for *π* (×, point at which the numerical optimization procedure converged): (a) mover–stayer gamma; (b) mover–stayer IG; (c) mover–stayer CP; (d) ZINB; (e) ZIPIG

Fig. 1 shows plots of the profile log‐likelihoods for *π*. From Fig. 1, the profile log‐likelihood for the Poisson mover–stayer gamma model is seen to be a monotonically decreasing function, which implies that the maximum is attained at the boundary, in particular at *π*=0, and not at the value that is produced from the numerical optimization procedure (reported in Table 1). The Poisson mover–stayer gamma model therefore suggests the non‐existence of a stayer population. As the optimization procedure did not converge at the maximum, a more relevant confidence interval (as opposed to a Wald interval) can be computed from the profile likelihood based on values of *π* in which the null hypothesis $H_{0}:\pi =0$ cannot be rejected. Such an interval was calculated as (0,0.063). The optimization routine for the other models (Poisson mover–stayer IG and CP models and zero‐inflated models) seem to have converged at the maximums of their respective profile log‐likelihoods. Furthermore, as the stayer proportions and their respective confidence intervals are estimated far from zero, these models are consistent with a stayer population. A likelihood ratio test of $H_{0}:\pi =0$ (as described in Section 3.3) resulted in *p*<0.001 for the Poisson mover–stayer IG and zero‐inflated models therefore indicating convincing evidence for a stayer population. From Table 1, it can, however, be seen that the stayer proportion estimates are widely varying across these models.

### 3.5 Model performance

Because the fitted models are non‐nested, standard asymptotic theory does not support formal hypothesis testing through comparison of likelihood values. Nevertheless, the zero‐inflated models are seen to have vastly larger likelihood values than the Poisson mover–stayer models. This would indicate, informally, that the zero‐inflated models provide a much improved fit relative to the Poisson mover–stayer models. If, however, the goodness of fit to the data is of specific, standalone, interest (in addition to providing a means of model comparison), likelihood values are less informative. Therefore, we compare the observed and estimated increments of damaged joints. Let

$$e_{ij}\left(d\right)=\hat{P}\left(D_{ij}=d|z_{ij},{\hat{\Lambda }}_{ij}\right),d=0,1,\ldots ,$$

be the estimated probability that patient *i* develops *d* additional damaged joints between $t_{ij}$ and $t_{ij+1}$. Here ${\hat{\Lambda }}_{ij}$ and $\hat{\pi }$ are the maximum likelihood estimates that were obtained in Section 3.4. For the Poisson mover–stayer models, $D_{ij}$ is assumed to have a Poisson distribution conditional on $u_{i}$. To obtain values of $e_{ij}\left(d\right)$ for these models, $u_{i}$ is estimated by using the empirical Bayes estimate, specifically,

$$\begin{array}{cc} {\hat{u}}_{i} & =E(U_{i}|{\hat{\lambda }}_{0},\hat{\beta },\hat{\phi },z_{i},t_{i})\\ & =\int_{0}^{\infty }u_{i}f\left(u_{i}|{\hat{\lambda }}_{0},\hat{\beta },\hat{\phi },z_{i},t_{i}\right)du_{i} \end{array}$$

where $\hat{\phi }$ is the maximum likelihood estimate for the set of parameters of the random‐effects distribution and $f(u_{i}|{\hat{\lambda }}_{0},\hat{\beta },\hat{\phi },z_{i},t_{i})$ denotes the predictive distribution of $u_{i}$, which takes the form

$$f\left(u_{i}|{\hat{\lambda }}_{0},\hat{\beta },\hat{\phi },z_{i},t_{i}\right)=\frac{L_{i}\left({\hat{\lambda }}_{0},\hat{\beta }|u_{i};z_{i}\right)g_{M-S}\left(u_{i}|\hat{\phi }\right)}{\int_{0}^{\infty }L_{i}\left({\hat{\lambda }}_{0},\hat{\beta }|u_{i};z_{i}\right)g_{M-S}\left(u_{i}|\hat{\phi }\right)du_{i}}.$$

Here $L_{i}\left({\hat{\lambda }}_{0},\hat{\beta }|u_{i};z_{i}\right)$ is the conditional likelihood contribution from the *i*th patient. For the zero‐inflated models, $D_{ij}$ is assumed to have a Poisson distribution conditional on $u_{ij}$ and $c_{i}$. Hence $D_{ij}$ has a negative binomial (NB) or Poisson IG distribution conditional on $c_{i}$ if $u_{ij}$ is drawn from a gamma or IG distribution respectively. Values of $e_{ij}\left(d\right)$ can then be obtained by using these marginal distributions and estimating $c_{i}$ by its empirical Bayes estimate, i.e.

$${\hat{c}}_{i}=\frac{\left(1-{\hat{\pi }}_{i}\right)L_{i}\left({\hat{\lambda }}_{0},\hat{\beta }|c_{i}=1;z_{i}\right)}{\left(1-{\hat{\pi }}_{i}\right)L_{i}\left({\hat{\lambda }}_{0},\hat{\beta }|c_{i}=1;z_{i}\right)+{\hat{\pi }}_{i}L_{i}\left({\hat{\lambda }}_{0},\hat{\beta }|c_{i}=0;z_{i}\right)}.$$

The observed and estimated changes in joint counts are displayed in Table 2. It is evident from Table 2 that none of the Poisson mover–stayer models provide particularly close agreements between the observed and estimated values. The category with increments of one damaged joint is considerably overestimated by all three models which then results in the majority of categories with larger increments of damaged joints being severely underestimated. However, the zero‐inflated models demonstrate much closer agreement, as also suggested by the likelihood values, which may indicate that time varying unobserved heterogeneity is strongly present in the data. The category corresponding to an increment of one damaged joint is overestimated to a much lesser degree by these models and as a result categories with larger incremental damage are underestimated less severely. One general statistic that accounts for the overall model performance at each category is the Pearson statistic. Let *o*(*d*) denote the observed number of times where *d* incremental damaged joints occurred and let $e\left(d\right)=\Sigma_{i}\Sigma_{j}e_{ij}\left(d\right)$. A Pearson statistic can then be defined as $\Sigma_{d}{\left\{o\left(d\right)-e\left(d\right)\right\}}^{2}/e\left(d\right)$. This statistic was calculated as 221.49, 203.46 and 256.52 for the fitted Poisson mover–stayer gamma, Poisson mover–stayer IG and Poisson mover–stayer CP models and 24.94 and 43.51 for the fitted ZINB and ZIPIG models respectively.

**Table 2:** Observed and estimated changes in joint counts from the Poisson mover–stayer and zero‐inflated models†

*Increments of*	*Observed*	*Results for the following models:*				
*damaged joints*						
		*Poisson mover–*	*Poisson mover–*	*Poisson mover–*	*ZINB*	*ZIPIG*
		*stayer gamma*	*stayer IG*	*stayer CP*		
0 without previous	6044	5974.94 (0.8)	5987.57 (0.53)	5954.33 (1.35)	5974.52 (0.81)	5974.94 (0.8)
damage						
0 with previous	2032	1871.25 (13.81)	1888.21 (10.95)	1861.51 (15.61)	2057.45 (0.31)	2050.72 (0.171)
damage						
1	250	528.89 (147.06)	505.29 (128.98)	559.2 (170.97)	310.09 (11.64)	337.3 (22.6)
2	97	91.97 (0.27)	87.07 (1.13)	94.92 (0.05)	88.39 (0.84)	76.84 (5.29)
3	28	27.24 (0.02)	26.31 (0.11)	26.94 (0.042)	37.48 (2.4)	31.83 (0.46)
4	26	11.62 (17.77)	11.36 (18.85)	11.29 (19.16)	19.37 (2.27)	16.84 (4.98)
5	17	6.3 (18.14)	6.17 (19.03)	6.08 (19.58)	11.33 (2.83)	10.17 (4.58)
6	8	3.97 (4.08)	3.87 (4.41)	3.8 (4.63)	7.23 (0.08)	6.69 (0.26)
7	6	2.77 (3.78)	2.69 (4.07)	2.63 (4.32)	4.91 (0.24)	4.66 (0.38)
8	7	2.08 (11.68)	2.04 (12.08)	1.97 (12.79)	3.51 (3.48)	3.39 (3.83)
>8	15	8.97 (4.06)	9.42 (3.3)	7.33 (8.02)	15.72 (0.03)	16.61 (0.16)
Total	8530	8530 (221.49)	8530 (203.46)	8530 (256.52)	8530 (24.94)	8530 (43.51)

†The estimated changes of *d* joint counts are calculated as $e\left(d\right)=\Sigma_{i}\Sigma_{j}e_{ij}\left(d\right)$. In parentheses are the Pearson statistic contributions. These are obtained by squaring the difference between the observed and estimated changes and then dividing by the estimated changes.

The Pearson statistic distribution under the null hypothesis that the fitted models are the data‐generating mechanisms can be used to assess more formally the goodness of fit of the fitted models. The exact form of this distribution is obtained from summing the independent but non‐identically distributed $e_{ij}\left(d\right)$s which are of a multinomial form. This, however, results in an intractable distribution. Alternatively, this distribution can be estimated by using a parametric bootstrap algorithm such as that described in Aguirre‐Hernández and Farewell (2004), in which a bootstrap data set *s* was simulated by first simulating $c_{i}^{s}$ from a Bernoulli distribution with success probability $1-\hat{\pi }$ for each patient. If the realized value $c_{i}^{s}$ was 0, the patient was a simulated stayer and therefore the simulated values $d_{ij}^{s}$ were 0 for all observed time intervals for this patient. Otherwise, each value $d_{ij}^{s}$ was simulated given ${\hat{\Lambda }}_{ij}$ and ${\hat{\theta }}^{\mathrm{nb}}$ from an NB or ${\hat{\psi }}^{\mathrm{pig}}$ from a Poisson IG model. To simulate this $d_{ij}^{s}$ from these distributions, a value $u_{ij}^{s}$ from either a gamma or IG distribution with parameter ${\hat{\theta }}^{\mathrm{nb}}$ or ${\hat{\psi }}^{\mathrm{pig}}$ is first sampled; then $d_{ij}^{s}$ is drawn from a Poisson distribution with mean $u_{ij}^{s}{\hat{\Lambda }}_{ij}$. The Pearson statistic can then be calculated for the bootstrap data set by refitting the same model structure onto this data set and calculating the corresponding $o^{s}\left(d\right)$ and $e^{s}\left(d\right)$ for all *d*.

This procedure was repeated 1000 times, hence producing 1000 realizations of the Pearson statistic under the null hypothesis that the fitted model is the underlying data‐generating mechanism. The *p*‐values for the models proposed are then calculated as being the proportion of these realizations greater than the Pearson statistic evaluated on the observed data set. The *p*‐values for the ZINB and ZIPIG models were calculated as 0.098 and less than 0.001 with the means of the bootstrap goodness‐of‐fit distribution being 16.56 and 15.77 respectively. These results suggest that there is little evidence of lack of agreement between the observed and estimated incremental joint damage from the ZINB model. However, there is more evidence of lack of agreement from the ZIPIG model.

## 4 Model features influencing estimation of *π*

In the previous sections, and as is commonly done in the literature, the parameter *π* was used to investigate the existence and proportion of a stayer population as it represents a statistically coherent framework. For example, one could test $H_{0}:\pi =0$ or examine its estimated confidence interval. In regard to the PA data, widely varying estimates of this parameter resulted when different models for the movers were specified. This, at the very minimum, would suggest a single analysis of a stayer population through *π* should not be adopted uncritically and that greater consideration of this parameter would be worthwhile. This section considers why different values of *π* may have resulted by investigating the interpretability of such a parameter across the fitted models.

Across the fitted models, it would be natural to postulate that patients who are highly likely or unlikely to be stayers have similar stayer probability estimates and that the main discrepancies from these estimates have resulted from disentangling slow transitioning movers and stayers. This may suggest, because of the widely varying estimates of *π*, that the PA data contained some patients who were slow transitioning movers and that the fitted models distinguished very differently between slow transitioning movers and stayers. If this is so, it would be intuitive to examine features of the fitted models, especially the model structure for the movers which generate the possibility and proportion of slow transitioning movers.

In the motivating application, both the Poisson mover–stayer IG and CP models strongly suggest the existence of a stayer population. For these models, the continuous part of the patient level random‐effects densities are such that $g(u_{i})\to 0$ when $u_{i}\to 0$, regardless of the parameter *ψ* (IG), and $g\left(u_{i}\right)\to \rho \nu \text{exp}\left(-\rho \right)$ as $u_{i}\to 0$ (CP). Thus it appears less likely that these distributions can place a large proportion of mass arbitrarily close to zero. If a slow transitioning mover population exists, the Poisson mover–stayer IG and CP models may therefore struggle to represent these patients adequately in the model for the movers, and hence these models may attribute slow transitioning movers with high stayer probabilities instead, i.e. the stayer proportions will be overestimated. Regarding the CP distribution, the parameter *ρ*, as discussed, governs both the overall baseline transition intensity *ρ*/*ν* and the stayer proportion  exp (−*ρ*). An overall slow or fast transition intensity, e.g. when there are many or few slow transitioning movers, as indicated by *ρ* will then also enforce a high or low stayer probability (through *ρ*) even if few or many stayers exist. This is not so for the other models as a slow or fast overall baseline transition intensity $\left(1-\pi \right)\lambda_{0}$ through $\lambda_{0}$ will not force *π* to take a certain value.

The class of zero‐inflated models also strongly suggests the existence of a stayer population. These models, unlike the others, do not contain patient level random effects and therefore are less able to represent specific patients with constantly low propensity of developing damage, after adjusting for covariates. Hence, for these zero‐inflated models, representing an arbitrary number of slow transitioning movers may also be problematic, particularly more so than with patient level random‐effect models, and this will again probably result in slow transitioning movers being attributed high stayer probabilities.

Of the fitted models, only the Poisson mover–stayer gamma model suggests the non‐existence of a stayer population since $\hat{\pi }=0$. This model was constructed by using patient level gamma random effects in the model for the movers. Under the current parameterization for $U_{i}\Lambda_{ij}$, if *θ*>1, the gamma distribution is such that $g(u_{i})\to \infty$ as $u_{i}\to 0$, and therefore this distribution can place a large proportion of mass arbitrarily close to zero. Implicitly, the gamma distribution can represent a large proportion of patients with small transition intensities, namely the slow transitioning movers. In contrast with the other models, the Poisson mover–stayer gamma model would seem to be at less risk of overestimating the stayer proportion; the stayer probabilities for the slow transitioning movers are less likely to be overestimated. However, if sufficient follow‐up information is not available, it is clear that identifiability issues will arise because stayers can be reasonably represented either through *π* or the left‐hand tail of the gamma distribution. In the motivating application, the fitted Poisson mover–stayer gamma model was such that $\hat{\theta }=6.19$ (5.11,7.5) with a small average estimated transition intensity. Thus this model seems to have inferred a slow transitioning mover population, instead of attributing these patients as stayers.

In summary, although *π* is defined as those with no propensity to develop damage (i.e. *λ*=0), this parameter seems to be heavily dependent on the model for the movers, at least for the fitted models, perhaps because it generates the possibility and proportion of slow transitioning movers. At a minimum, this would suggest that a more appropriate interpretation of *π* would be the proportion of patients who are at no or at least minimal risk of damage, which cannot be adequately explained by the fitted model for the movers, although the fitted model for the movers may not necessarily fit well. This would be particularly relevant for the motivating application, especially when considering the differences between the Poisson mover–stayer gamma model and the others, because the model structures for the movers specified vastly different probability distributions for the risk of damage.

### 4.1 Simulation study

To formalize ideas and to investigate estimation of *π* further by using the considered (and commonly advocated) models, we perform some simulation studies. These studies will represent the scenarios where there are few or many slow transitioning movers in addition to a stayer population. In the first scenario, where few patients are slow transitioning movers, a lesser distinction between estimated values of *π* is expected, although possibly not for the Poisson mover–stayer CP model. In the second scenario, where many slow transitioning movers exists, there should be a greater distinction between the estimates of *π*, specifically between the Poisson mover–stayer gamma model and the other models which are less able to represent an arbitrary number of slow transitioning movers through the left‐hand tail of their mover distribution.

In scenario 1, we simulate from the Poisson mover–stayer gamma, Poisson mover–stayer IG, Poisson mover–stayer CP, ZINB and ZIPIG models with no covariates and {*π*=0.3,*λ*=0.3,*θ*=4}, {*π*=0.3,*λ*=0.5,*ψ*=1}, {*π*= exp (−*ρ*)=0.3,*ν*=4}, $\left\{\pi =0.3,\lambda =0.3,\theta^{\mathrm{nb}}=4\right\}$ and $\left\{\pi =0.3,\lambda =0.3,\psi^{\mathrm{pig}}=0.5\right\}$ respectively. In scenario 2, we simulate from the same models with no covariates and {*π*=0.3,*λ*=0.5,*θ*=1}, {*π*=0.3,*λ*=0.5,*ψ*=0.5}, {*π*= exp (−*ρ*)=0.3,*ν*=1}, $\left\{\pi =0.3,\lambda =0.15,\theta^{\mathrm{nb}}=4\right\}$ and $\left\{\pi =0.3,\lambda =0.15,\psi^{\mathrm{pig}}=0.5\right\}$ respectively. Smaller values of *λ* and/or greater values of *θ*, 1/*ψ* and *ν* were chosen in scenario 2 to generate the setting where there would be more slow transitioning movers than in scenario 1. Simulations from the Poisson mover–stayer models were performed by first simulating $u_{i}^{s}$ for each patient. The damage joints increments $d_{ij}^{s}$ were then simulated from a Poisson distribution with mean $u_{i}^{s}\Lambda_{ij}$. For the Poisson mover–stayer gamma and IG models, $u_{i}^{s}$ was obtained from $c_{i}^{s}v_{i}^{s}$ where $c_{i}^{s}$ is a realization from a Bernoulli distribution with success probability 1−*π* and $v_{i}^{s}$ is a realization from either a gamma or IG distribution with mean 1 and variance *θ* or 1/*ψ* respectively. For the Poisson mover–stayer CP model, $u_{i}^{s}$ was obtained by simulating from a gamma distribution with rate *ν* and shape $n_{i}^{s}$ where $n_{i}^{s}$ is generated from a Poisson distribution with mean *ρ*. Simulations from the zero‐inflated models were performed by simulating $d_{ij}^{s}$ from a Poisson distribution with mean $c_{i}^{s}u_{ij}^{s}\Lambda_{ij}$ where $c_{i}^{s}$ is as before and $u_{ij}^{s}$ is a realization from either a gamma or IG distribution with mean 1 and variance $\theta^{\mathrm{nb}}$ or $1/\psi^{\mathrm{pig}}$ respectively.

In both scenarios, 1000 data sets were simulated with each containing 200 patients. Each patient was assumed to have 11 clinic visits annually. The Poisson mover–stayer and zero‐inflated models were then fitted to each data set.

Tables 3 and 4 display the results of the simulation study under scenario 1 and scenario 2 respectively. Only results on estimates of *π* are displayed although the baseline intensities and dispersion parameters were also estimated. In both scenarios, there were some similar characteristics to those seen in the motivating application. In particular, the zero‐inflated models generally provide the highest stayer proportion estimates, and furthermore the estimates were similar across the ZINB and ZIPIG models. However, across both scenarios, it is clear that the stayer proportion estimates are considerably more biased in scenario 2, as indicated by the difference in stayer proportion estimates from each model and the true model (the third column in Tables 3 and 4). This is especially so when the Poisson mover–stayer gamma and IG models are the true model. These results suggest that the model for the movers is particularly influential in the estimation of *π* when there are many slow transitioning movers. Indeed, no model, on average, could accurately estimate the true stayer proportion in all the settings in scenario 2. A noteworthy observation can be made when the zero‐inflated models are the true models. In this case, the Poisson mover–stayer CP model estimates considerably smaller stayer proportions than the other models which are in close agreement with one another. The zero‐inflated models in these simulation settings cannot generate patient‐specific propensities for damage since patient level random effects or dynamic covariates are not included in these models. Moreover, they can generate only independent observation‐specific propensities of gaining damage. Therefore, there is a lesser need for the patient level random effects in the Poisson mover–stayer models, which is reflected through a larger estimated value of $\hat{\nu }$ (equivalently the continuous part of the CP distribution is estimated to have smaller variance) in the Poisson mover–stayer CP model. If the overall baseline intensity in the Poisson mover–stayer CP model, $\hat{\rho }/\hat{\nu }$, is to be similar to the other models, $\hat{\rho }$ will then be required to take a larger value. This causes the estimated stayer proportion $\text{exp}(-\hat{\rho })$ to take a smaller value.

**Table 3:** Results of the simulation study under scenario 1†

*Model*	$\hat{\pi }\;\left(SE,\;SD\right)$	$\hat{\pi }-{\hat{\pi }}_{\;P\;M-S\;\mathrm{True}}$	*% of simulations*
		(*SD*)	*where* $\hat{\pi }\approx 0$
*Poisson mover–stayer gamma* (*π*=0.3,*λ*=0.5,*θ*=1)			
Poisson mover–stayer gamma	0.29 (0.068, 0.07)	—	0.5
Poisson mover–stayer IG	0.36 (0.041, 0.042)	0.071 (0.039)	0
Poisson mover–stayer CP	0.31 (0.04, 0.04)	0.021 (0.06)	0
ZINB	0.41 (0.035 0.035)	0.12 (0.055)	0
ZIPIG	0.41 (0.035, 0.035)	0.12 (0.055)	0
*Poisson mover–stayer IG* (*π*=0.3,*λ*=0.5,*ψ*=1)			
Poisson mover–stayer gamma	0.21 (0.076, 0.077)	−0.091 (0.048)	3.2
Poisson mover–stayer IG	0.3 (0.043, 0.043)	—	0
Poisson mover–stayer CP	0.26 (0.038, 0.038)	−0.043 (0.028)	0
ZINB	0.36 (0.034 0.034)	0.063 (0.019)	0
ZIPIG	0.36 (0.034, 0.034)	0.063 (0.019)	0
*Poisson mover–stayer CP* (*π*=0.3,*ν*=1)			
Poisson mover–stayer gamma	0.3 (0.037, 0.037)	0.002 (0.018)	0
Poisson mover–stayer IG	0.31 (0.034, 0.034)	0.028 (0.008)	0
Poisson mover–stayer CP	0.3 (0.034, 0.031)	—	0
ZINB	0.34 (0.03, 0.033)	0.034 (0.006)	0
ZIPIG	0.34 (0.033, 0.033)	0.034 (0.006)	0
*ZINB* $\left(\pi =0.3,\lambda =0.3,\theta^{\mathrm{nb}}=4\right)$			
Poisson mover–stayer gamma	0.3 (0.056, 0.056)	0.004 (0.01)	0
Poisson mover–stayer IG	0.33 (0.045, 0.045)	0.023 (0.017)	0
Poisson mover–stayer CP	0.18 (0.042, 0.044)	−0.12 (0.017)	0
ZINB	0.3 (0.044, 0.044)	—	0
ZIPIG	0.3 (0.044, 0.044)	0.005 (0.001)	0
*ZIPIG* $\left(\pi =0.3,\lambda =0.3,\psi^{\mathrm{pig}}=0.5\right)$			
Poisson mover–stayer gamma	0.3 (0.04, 0.04)	−0.003 (0.015)	8.2
Poisson mover–stayer IG	0.3 (0.04, 0.04)	−0.002 (0.013)	0
Poisson mover–stayer CP	0.12 (0.038, 0.039)	−0.18 (0.013)	0
ZINB	0.3 (0.039, 0.037)	−0.001 (0.001)	0
ZIPIG	0.3 (0.039, 0.04)	—	0

†The second column displays the mean and standard deviation SD of the estimated values of *π* together with the mean standard error SE. The third column displays the mean and standard deviation of the estimated difference between *π* from each model and *π* from the true model. The fourth column displays the percentage of simulations where $\hat{\pi }\approx 0$.

**Table 4:** Results of the simulation study under scenario 2†

*Model*	$\hat{\pi }\;\left(SE,\;SD\right)$	$\hat{\pi }-{\hat{\pi }}_{\;P\;M-S\;\mathrm{true}}$	*% of simulations*
		(*SD*)	*where* $\hat{\pi }\approx 0$
*Poisson mover–stayer gamma* (*π*=0.3,*λ*=0.3,*θ*=4)			
Poisson mover–stayer gamma	0.28 (0.23, 0.22)	—	28.7
Poisson mover–stayer IG	0.59 (0.056, 0.056)	0.3 (0.18)	0
Poisson mover–stayer CP	0.61 (0.039, 0.039)	0.33 (0.21)	0
ZINB	0.67 (0.034, 0.033)	0.38 (0.21)	0
ZIPIG	0.67 (0.034, 0.033)	0.38 (0.21)	0
*Poisson mover–stayer IG* (*π*=0.3,*λ*=0.3,*ψ*=0.5)			
Poisson mover–stayer gamma	0.061 (0.086, 0.085)	−0.23 (0.065)	58.9
Poisson mover–stayer IG	0.3 (0.055, 0.055)	—	0
Poisson mover–stayer CP	0.33 (0.039, 0.039)	0.37 (0.42)	0
ZINB	0.41 (0.035, 0.035)	0.11 (0.33)	0
ZIPIG	0.41 (0.035, 0.035)	0.11 (0.34)	0
*Poisson mover–stayer CP* (*π*=0.3,*ν*=4)			
Poisson mover–stayer gamma	0.31 (0.064, 0.062)	0.01 (0.05)	0
Poisson mover–stayer IG	0.37 (0.042, 0.041)	0.07 (0.22)	0
Poisson mover–stayer CP	0.3 (0.041, 0.04)	—	0
ZINB	0.42 (0.035, 0.035)	0.12 (0.011)	0
ZIPIG	0.42 (0.035, 0.034)	0.12 (0.011)	0
*ZINB* $\left(\pi =0.3,\lambda =0.15,\theta^{\mathrm{nb}}=4\right)$			
Poisson mover–stayer gamma	0.28 (0.12, 0.12)	−0.016 (0.1)	9.6
Poisson mover–stayer IG	0.32 (0.085, 0.084)	0.029 (0.06)	2.6
Poisson mover–stayer CP	0.16 (0.057, 0.058)	−0.13 (0.035)	0
ZINB	0.29 (0.069, 0.07)	—	0
ZIPIG	0.3 (0.068, 0.07)	0.005 (0.061)	0
*ZIPIG* $\left(\pi =0.3,\lambda =0.15,\psi^{\mathrm{pig}}=0.5\right)$			
Poisson mover–stayer gamma	0.27 (0.1, 0.1)	−0.024 (0.08)	19.5
Poisson mover–stayer IG	0.29 (0.084, 0.08)	−0.076 (0.062)	8.2
Poisson mover–stayer CP	0.091 (0.047, 0.048)	−0.2 (0.03)	0
ZINB	0.29 (0.062, 0.064)	−0.002 (0.002)	0
ZIPIG	0.29 (0.061, 0.063)	—	0

†The column headings have the same interpretation as those stated in the footnote to Table 3.

When the Poisson mover–stayer gamma and IG models are the true models in scenario 2, Table 4 indicates that the Poisson mover–stayer gamma model has estimated $\hat{\pi }\approx 0$ in 28.7% and 58.9% of simulated data sets respectively, whereas none of the other models estimate $\hat{\pi }\approx 0$ for any of the same simulated data sets. Similarly, when the zero‐inflated models are the true model in scenario 2, the Poisson mover–stayer gamma model provides the greatest proportion of estimates where $\hat{\pi }\approx 0$. As discussed, the Poisson mover–stayer gamma model has greater ability to represent an arbitrary number of slow transitioning movers, and thus more information, e.g. in the form of longer follow‐up or a smaller proportion of slow transitioning movers (as in scenario 1), is required for the Poisson mover–stayer gamma model to identify a non‐zero stayer proportion, if one exists, when compared with the other models. Conversely, as indicated in scenario 2, particularly when the Poisson mover–stayer gamma model is the true model, other models may greatly overestimate the stayer proportion with a relatively narrow confidence interval when many slow transitioning movers are contained in the data. This observation was also verified even when no stayer population exists (results from this simulation study are not shown). Overall, these results emphasize the difficulty of estimating a stayer proportion when many slow transitioning movers exists. Models which are more able to represent an arbitrary number of slow transitioning movers may be the most appropriate in estimating the stayer proportion, because they are at less risk of overestimating the stayer proportion. But with less informative data (because of many slow transitioning movers and limited follow‐up) these models may suggest, in the extreme case, that $\hat{\pi }\approx 0$ even if a stayer population exists, as seen in scenario 2 where *π*=0.3. Models which are less able to represent slow transitioning movers require significantly less informative data to identify a non‐zero stayer proportion; however, such an estimate may be an overestimate of the true stayer proportion in the presence of slow transitioning movers.

## 5 Patient and observation level random‐effects models

### 5.1 Model description

The simulation studies demonstrated that widely varying stayer proportion estimates, as seen in the motivating application, may result from the choice of structure and distributional assumption of the random effects, the existence of many slow transitioning movers and lack of information in the data. Nevertheless, the lack of fit with the Poisson mover–stayer models provides an additional complicating factor in interpreting the models for this application. As the zero‐inflated models provide a much improved fit to the data, which suggests that time varying unobserved heterogeneity could be present in the PA data, a natural progression would be to provide this same adjustment for the Poisson mover–stayer models. This, as before, can be achieved through the incorporation of observation level random effects. If this approach is adopted, the patient level random effects will primarily be designed to introduce correlation between patient observations, as opposed to previously, where it was also designed to capture unobserved heterogeneity.

Let $U_{i}$ and $U_{ij}$ be multiplicative patient level mover–stayer and observation level random effects respectively. Assume that $D_{ij}$ is Poisson distributed with mean

$$u_{i}u_{ij}\Lambda_{ij}=u_{i}u_{ij}\left(t_{ij+1}-t_{ij}\right)\lambda_{0}\text{exp}\left(\beta^{\prime }z_{ij}\right).$$

Then, under the usual assumption that $U_{i}$ and $U_{ij}$ are independent, the marginal likelihood for patient *i* is

$${\left\{\;\;\int_{0}^{\infty }\prod_{j=0}^{m_{i}-1}h\left(d_{ij}|u_{i};\Lambda_{ij}\right)f\left(u_{i}\right)du_{i}\right\}}^{c_{i_{*}}}{\left\{\pi_{i}+\int_{0}^{\infty }\prod_{j=0}^{m_{i}-1}h\left(0|u_{i};\Lambda_{ij}\right)f\left(u_{i}\right)du_{i}\right\}}^{1-c_{i_{*}}}$$

where $c_{i_{*}}$ is as before and

$$h\left(d|u_{i};\Lambda_{ij}\right)=\int_{0}^{\infty }\frac{{\left(u_{ij}u_{i}\Lambda_{ij}\right)}^{d}\text{exp}\left(-u_{ij}u_{i}\Lambda_{ij}\right)}{d!}g\left(u_{ij}\right)du_{ij}.$$

These models were examined with the three patient level mover–stayer random‐effects distributions that were described in Section 3.1 and with the gamma distribution (with parameter $\theta^{\mathrm{nb}}$ as before) for the observation level random effects. The marginal likelihood was evaluated by first computing the integrations over the gamma observation level random effects, resulting in NB models conditional on $U_{i}$, then using the integrate command to perform the integrations over the patient level random effects. These models will be referred to as NB mover–stayer models, and further qualification, when needed, will again be through the addition of the type of patient level random‐effects distribution that is used. Note that the ZINB model is obtained from the NB mover–stayer gamma and NB mover–stayer IG models when *θ* and 1/*ψ*=0 respectively, whereas the class of Poisson mover–stayer models is obtained when $\theta^{\mathrm{nb}}=0$. The same explanatory variables are again considered for regression purposes.

### 5.2 Results

Table 5 displays the results from fitting the three NB mover–stayer models. Covariate effects estimated from this model structure also remain relatively stable across different random‐effects distributional assumptions. Furthermore, apart from the attained number of damaged joints, the covariate effects are comparable with those produced by the ZINB model. The number of damaged joints attained now demonstrates a much smaller negative effect size than in the Poisson mover–stayer models (when gamma observation level random effects are not included). This may have resulted because the attained number of damaged joints and the patient level random effects are more confounded than before; the patient level random effect is now primarily designed to introduce correlation between patient observations. This would also suggest that there is (at most) little effect of accounting for the number of joints at risk of damage for these models.

**Table 5:** Parameter estimates of associations with damaged joint counts and stayer probability estimates obtained from fitting the NB mover–stayer (patient and observation level random‐effects) models to 757 psoriatic arthritis patients

	*Results for the following models:*		
	*NB mover–*	*NB mover–*	*NB mover–*
	*stayer gamma*	*stayer IG*	*stayer CP*
Attained number of damaged joints	−0.036 (−0.07,−0.00087)	−0.053 (−0.098,−0.0084)	−0.0047 (−0.034,0.025)
Current number of active joints	0.087 (0.058, 0.12)	0.085 (0.055, 0.11)	0.093 (0.065, 0.12)
Arthritis duration	0.026 (0.0071, 0.044)	0.03 (0.0096, 0.051)	0.018 (0.0021, 0.034)
Age at onset of arthritis	0.0097 (−0.0062,0.026)	0.0093 (−0.0076,0.026)	0.0087 (−0.0051,0.023)
$\lambda_{0}$	0.058 (0.029, 0.12)	0.087 (0.041, 0.19)	1
$\theta^{\mathrm{nb}}$	6.52 (5.32, 8)	6.27 (5.07, 7.75)	7.17 (5.9, 8.71)
*θ*	3.11 (2.24, 4.33)		
*ψ*		0.31 (0.14, 0.7)	
*ν*			18.75 (9.78, 35.94)
*ρ*			1.06 (0.84, 1.34)
*π*	0.0025 (0, 1)	0.3 (0.18, 0.45)	0.34 (0.26, 0.43)
Log‐likelihood	−2249.91	−2249.9	−2253.89

**Figure 2:**
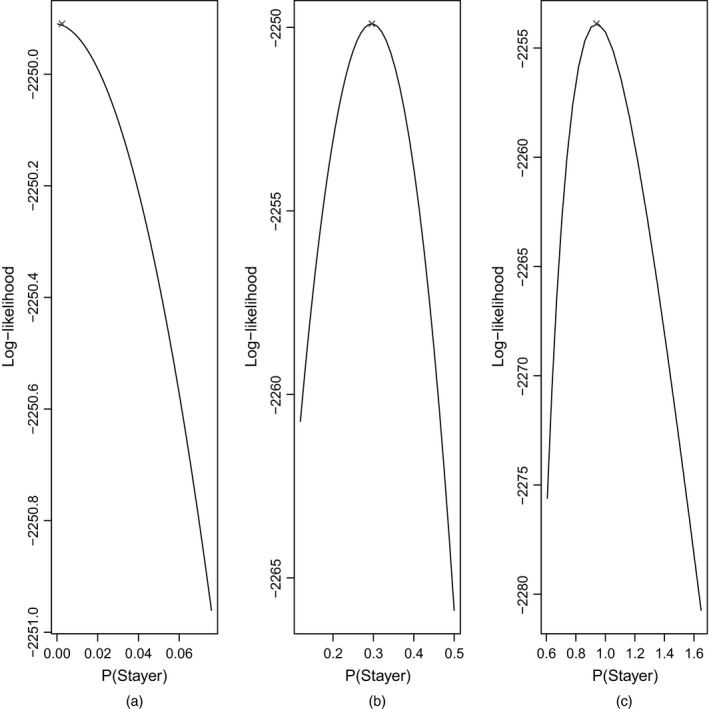
Plots of the profile log‐likelihoods for *π* (×, point at which the numerical optimization procedure converged): (a) NB mover–stayer gamma; (b) NB mover–stayer IG; (c) NB mover–stayer CP

Fig. 2, which displays the profile log‐likelihood for *π*, demonstrates that the numerical optimization routine converged at the maximum of the profile log‐likelihoods of the NB mover–stayer IG and NB mover–stayer CP models, but not for the NB mover–stayer gamma model. The estimated value of *π* from the NB mover–stayer IG and CP models were 0.3 (0.18,0.45) and 0.34 (0.26,0.43) respectively and are therefore in much closer agreement than when observation level random effects were not included, i.e. compared with the difference between the Poisson mover–stayer IG and Poisson mover–stayer CP models. Both models again provide strong evidence for the existence of a stayer population through the size and confidence interval of $\hat{\pi }$. A test of the null hypothesis $H_{0}:\pi =0$ for the NB mover–stayer IG model resulted in *p*<0.001, thus providing convincing evidence to reject this null hypothesis. The maximum of the profile log‐likelihood in Fig. 2 for the NB mover–stayer gamma model can again be seen as $\hat{\pi }=0$, with a 95% likelihood ratio interval calculated as (0,0.086). Even after accounting for time varying unobserved heterogeneity, the NB mover–stayer gamma model suggests the non‐existence of a stayer population and therefore its inferences through *π* are again vastly different from those from the NB mover–stayer IG and CP models.

A (generalized) likelihood ratio test of $H_{0}:\theta^{\mathrm{nb}}=0$ resulted in *p*<0.001 for each of the fitted NB mover–stayer models, therefore suggesting that it is necessary to account for time varying unobserved heterogeneity. The large decrease in log‐likelihood values when compared with the Poisson mover–stayer models also suggest a much improved fit to the data. A comparison of the observed and estimated incremental joint damage from these models now follows.

### 5.3 Comparison of the observed and estimated incremental joint damage

As in Section 3.5, $e_{ij}\left(d\right)$ can be obtained through

$$e_{ij}\left(d\right)=\frac{\Gamma (d+1/{\hat{\theta }}^{\mathrm{nb}})}{d\Gamma (1/{\hat{\theta }}^{\mathrm{nb}})}{\left(\frac{{\hat{u}}_{i}{\hat{\theta }}^{\mathrm{nb}}{\hat{\Lambda }}_{ij}}{1+{\hat{u}}_{i}{\hat{\theta }}^{\mathrm{nb}}{\hat{\Lambda }}_{ij}}\right)}^{d}{\left(\frac{1}{1+{\hat{u}}_{i}{\hat{\theta }}^{\mathrm{nb}}{\hat{\Lambda }}_{ij}}\right)}^{1/{\hat{\theta }}^{\mathrm{nb}}},d=1,\ldots ,$$

where ${\hat{\Lambda }}_{ij}$ and ${\hat{\theta }}^{\mathrm{nb}}$ are the maximum likelihood estimates obtained in the previous subsection and where ${\hat{u}}_{i}$ (which is a function of ${\hat{\pi }}_{i}$) is the empirical Bayes estimate of the random effect as described in Section 3.5.

Table 6 displays the observed and estimated incremental joint damage from the NB mover–stayer models. These models demonstrate a much improved fit to the data when compared with the Poisson mover–stayer models, which is consistent with the respective likelihood values. From Table 6, it is interesting to observe that these models provide similar agreements between estimated and observed increments of damaged joints across all categories. In particular, there is some evidence that all three models still overestimate the category corresponding to one incremental joint damage. Overall, the Pearson statistic was calculated as 37.07, 38.06 and 35.89 for the fitted NB mover–stayer gamma, IG and CP models respectively.

**Table 6:** Observed and estimated changes in joint counts from the NB mover–stayer models†

*Increments of*	*Observed*	*Results for the following models:*		
*damaged joints*				
		*NB mover–*	*NB mover–*	*NB mover–*
		*stayer gamma*	*stayer IG*	*stayer CP*
0 without previous	6044	5973.69 (0.83)	5972.84 (0.85)	5974.07 (0.82)
damage				
0 with previous	2032	2032.16 (1.3$\times 10^{-5}$)	2030.62 (0.00094)	2037.52 (0.015)
damage				
1	250	338.87 (23.09)	341.17 (24.36)	334.35 (21.28)
2	97	88.16 (0.91)	87.56 (1.02)	88.62 (0.79)
3	28	36.5 (1.96)	36.16 (1.84)	36.53 (1.99)
4	26	18.85 (2.73)	18.74 (2.81)	18.68 (2.86)
5	17	11.06 (3.2)	11.07 (3.17)	10.88 (3.45)
6	8	7.07 (0.12)	7.12 (1.07)	6.91 (0.17)
7	6	4.81 (0.3)	4.87 (0.26)	4.67 (0.38)
8	7	3.42 (3.75)	3.49 (3.52)	3.31 (4.11)
>8	15	15.42 (0.01)	16.34 (1.1)	14.47 (0.02)
Total	8530	8530 (37.07)	8530 (38.06)	8530 (35.89)

†The estimated changes of *d* joint counts are calculated as $e\left(d\right)=\Sigma_{i}\Sigma_{j}e_{ij}\left(d\right)$. In parentheses are the Pearson statistic contributions. These are obtained by squaring the difference between the observed and estimated changes and then dividing by the estimated changes.

## 6 Does a stayer population exist in the psoriatic arthritis data?

This paper was motivated by the desire to obtain empirical evidence to support the existence or non‐existence of a stayer population who cannot develop damaged hand joints. For this purpose, we fitted multiple mover–stayer models to a comprehensive psoriatic arthritis data set and analysed their inferences, performance and features. As a result, reasonable fitting models and greater understanding about the interpretation of inferences were obtained. Overall, such an analysis has instilled greater confidence in the conclusions drawn.

When there are many slow transitioning movers (relatively to the number of patients), the results of Section 4 indicate that estimating the stayer proportion can be problematic. Unfortunately, this may have been so for the psoriatic arthritis data since similar results were observed to those in scenario 2 of the simulation study. In particular, there were large discrepancies in the estimated values of *π* between the fitted models with patient level gamma random effects and the other fitted models. The primary difficulty results from the other fitted models (those where the distribution of the patient level random effects, if included, were not chosen to be gamma) being less able to represent an arbitrary number of slow transitioning movers, as discussed in Section 4, and therefore their estimated value of *π* may not represent only the proportion of stayers but also the slow transitioning movers. Although the models with patient level gamma random effects, which are less likely to overestimate the stayer proportion, may suggest the non‐existence of a stayer population even if a stayer population does exist; because *π* may become non‐identifiable for these models when there are many slow transitioning movers. This was seen in the simulation study under multiple settings from scenario 2, particularly when the Poisson mover–stayer IG model was the true model and 58.9% of the simulated data sets produced values of $\hat{\pi }\approx 0$ even though the true value was *π*=0.3. The widely varying stayer proportion estimates of 0 (0, 0.086), 0.31 (0.18, 0.45) and 0.34 (0.26, 0.43) from the NB mover–stayer gamma, IG and CP models respectively, which all provided reasonable fits to the psoriatic arthritis data, therefore suggest that there is inconclusive evidence to support the existence of a stayer population who cannot develop damaged hand joints. Indeed, longer follow‐up may demonstrate that the non‐zero stayer proportion estimates from the NB mover–stayer IG and CP models were in fact overestimates of the true stayer proportion, which may be 0, due to the slow transitioning movers, whereas the NB mover–stayer gamma model may have suggested the non‐existence of a stayer population, even if one truly did exist, because of identifiability issues due to lack of follow‐up information. It is, however, important to note that the NB mover–stayer models are in strong agreement regarding the existence of a minimal risk population, as suggested by the non‐zero estimated values of *π* and its corresponding confidence interval from the NB mover–stayer IG and CP models as well as the estimated shape of the gamma distribution ($\hat{\theta }=3.11$ (2.24,4.33)) from the NB mover–stayer gamma model. Such a population, in itself, may be of most clinical interest especially because these patients are at a lower risk of developing immediate damage. Identifying patients in this subset can then be facilitated through introducing covariates into the model for *π* within the reasonable fitting models, as discussed in Section 3.3.

## 7 Discussion

Mover–stayer counting process models are commonly used in the literature. In many instances, a single model is fitted (sometimes including the non‐mover–stayer version) and conclusions regarding a stayer population are drawn based solely on that model. This paper demonstrates that, when fitted to the same data, different mover–stayer models can lead to widely varying stayer proportion estimates even if all the models provide reasonable fits to the data, e.g. the fitted NB mover–stayer models in the motivating application. Thus, if such an analysis is adopted uncritically, specific conclusions on a stayer population may be reached even though different models fitted to the same data may produce vastly different results.

A model's ability to account adequately for slow transitioning movers is a worthwhile consideration when estimation of a stayer proportion is of interest, i.e. whether the model for the movers can adequately allow for an arbitrary number of patients who have constantly low propensity to experience the event of interest, if these patients exist. Section 4 provides a discussion for some of the most commonly used models. Models which are less able to account for an arbitrary number of slow transitioning movers may not be able to provide an accurate estimate of the stayer proportion, especially if there are many slow transitioning movers, because their estimated value of *π* may not only represent stayers, as intended, but also patients who have a minimal risk of the event of interest (which is not adequately accounted for in the model for the movers). Fitting multiple mover–stayer models which are more and less able to account for many slow transitioning movers can therefore contribute greater understanding or confidence regarding the composition of stayers and slow transitioning movers, which will probably be of clinical interest in many cases since neither are likely to develop immediate damage. The simulation study demonstrated a potential pitfall regarding the use of mover–stayer models on less informative data, e.g. because of relatively short follow‐up, particularly the possibility that these models suggest the non‐existence of a stayer population even if such a population exists. Such an observation may be seen as counterintuitive if the lack of sufficient information in the data is thought to manifest itself through large standard errors as opposed to greatly altering the point estimate. This consideration may therefore be useful, especially if it leads to more appropriate interpretation of $\hat{\pi }$. It should be noted, as was clearly seen in the simulation study, that models which are more able to represent an arbitrary number of slow transitioning movers will probably require more informative data to suggest a non‐zero stayer proportion, and therefore this potential pitfall will be most relevant for these models such as those with patient level gamma random effects. Additionally, it is useful to examine model goodness of fit as this facilitates understanding important characteristics of the distribution of events in the movers. Accounting for these characteristics, such as observation level unobserved heterogeneity, may result in more reasonable fitting models therefore instilling greater confidence in the inferences obtained, and may also provide more similar estimates of *π* between competing models.

This work considered practical models that can be fitted reasonably easily, because of closed form marginal likelihoods or marginal likelihoods that can be evaluated by using a single integration per patient, that accommodated important characteristics of the data, such as within‐patient correlation and time varying unobserved heterogeneity. Thus this work considered models that have commonly been used, or are likely to be used in the future, to demonstrate potential pitfalls that may occur if a single analysis regarding the existence of a mover–stayer scenario is adopted uncritically. A natural extension would consist of developing more flexible models, e.g. by using random‐effects structures that account for time varying unobserved heterogeneity, such as those proposed in Aalen *et al*. (2008) and Unkel *et al*. (2014). Most of these random‐effects structures when used in conjunction with intermittently observed Poisson processes and where the follow‐up is extensive, such as in the psoriatic arthritis data, will, however, result in an intractable marginal likelihood, and therefore further research will probably be required. This work also did not focus on predicting a new patient's outcomes but instead focused on understanding the outcomes from a specific set of patients. Thus the goodness‐of‐fit test proposed, which utilized in‐sample prediction, did not penalize a model for overfitting the data. More appropriate model assessment criteria for prediction, if it is of interest, would involve out‐of‐sample prediction or other forms of penalty functions to identify overly complex models.

The results in this paper suggest that the models fitted, taken together, do not provide conclusive evidence for the existence of a stayer population who cannot develop damaged hand joints. However, there is convincing evidence of a subpopulation of patients who are at least at minimal risk of damage, where minimal risk is characterized by the fitted models. An interesting area of future work, at least from a clinical perspective, would be to characterize minimal risk directly on the basis of clinical considerations, perhaps through specifying a threshold for the damage progression rate, and then to develop models which can estimate the proportion of patients who belong to this user‐specified characterization. At a minimum, this will ensure that the target of inference will be of clinical interest and perhaps estimation of *π* will be less model dependent because *π* will be characterized by using a range of values of the damage progression rate and not based solely on zero.

## Supporting information

‘Supporting material for “Exploring the existence of a stayer population with mover–stayer counting process models: Application to joint damage in psoriatic arthritis”’.
