# Supplementary material for: Exploring the existence of a stayer population with mover–stayer counting process models: application to joint damage in psoriatic arthritis
Source: J R Stat Soc Ser C Appl Stat. 2016 Oct 19;66(4):669–90. doi: 10.1111/rssc.12187 (PMC5503139; doi:10.1111/rssc.12187)
Supplement: Supplementary file 1 — ‘Supporting material for “Exploring the existence of a stayer population with mover–stayer counting process models: Application to joint damage in psoriatic arthritis”’. [file RSSC-66-669-s001.pdf]

Supplementary material for "Exploring the existence of a stayer population with mover-stayer counting process models: Application to joint damage in psoriatic arthritis"

Sean Yiu*, Vernon T. Farewell, Brian D.M. Tom
*Email-address: sean.yiu@mrc-bsu.cam.ac.uk
MRC Biostatistics Unit, Cambridge, CB2 0SR, UK

Web Appendix A

To compute the marginal likelihoods in Section 3, the unobserved random effects have to be integrated out. That is the following integral must be evaluated

$$\int_0^\infty \prod_{j=0}^{m_i-1} \frac{(u_i\Lambda_{ij})^{d_{ij}} \exp\left(-u_i\Lambda_{ij}\right)}{d_{ij}!} g(u_i)\, du_i$$

where $g(u_i)$ is the density function of the unobserved random effects. Derivation of the mixture densities for the cases where $g(u_i)$ is the density function of a gamma, IG and the continuous part of the CP distribution is what follows. The following notation is used throughout, $d_i = \sum_j d_{ij}$ and $\Lambda_i = \sum_j \Lambda_{ij}$.

Gamma

$$\begin{aligned}
&\int_0^\infty \prod_{j=0}^{m_i-1} \frac{\Lambda_{ij}^{d_{ij}}}{d_{ij}!} \exp\left(-u_i\Lambda_{ij}\right) \frac{\left(\frac{1}{\theta}\right)^{\frac{1}{\theta}}}{\Gamma\left(\frac{1}{\theta}\right)} u_i^{d_i+\frac{1}{\theta}-1} \exp\left(-\frac{u_i}{\theta}\right)\, du_i \\
&= \left(\prod_{j=0}^{m_i-1} \frac{\Lambda_{ij}^{d_{ij}}}{d_{ij}!}\right) \frac{\left(\frac{1}{\theta}\right)^{\frac{1}{\theta}}}{\Gamma\left(\frac{1}{\theta}\right)} \int_0^\infty \frac{\Gamma\left(d_i+\frac{1}{\theta}\right)}{\left(\frac{1}{\theta}+\Lambda_i\right)^{d_i+\frac{1}{\theta}}} \frac{\left(\frac{1}{\theta}+\Lambda_i\right)^{d_i+\frac{1}{\theta}}}{\Gamma\left(d_i+\frac{1}{\theta}\right)} u_i^{d_i+\frac{1}{\theta}-1} \exp\left(-u_i\left(\frac{1}{\theta}+\Lambda_i\right)\right)\, du_i \\
&= \left(\prod_{j=0}^{m_i-1} \frac{\Lambda_{ij}^{d_{ij}}}{d_{ij}!}\right) \frac{\Gamma\left(d_i+\frac{1}{\theta}\right)}{\Gamma\left(\frac{1}{\theta}\right)} \frac{\theta^{d_i}}{\left(1+\theta\Lambda_i\right)^{d_i+\frac{1}{\theta}}}.
\end{aligned}$$

IG

$$\left(\prod_{j=0}^{m_i-1}\frac{\Lambda_{ij}^{d_{ij}}}{d_{ij}!}\right)\int_0^\infty u_i^{d_i}\exp(-\Lambda_i u_i)\left(\frac{\psi}{2\pi u_i^3}\right)^{\frac{1}{2}}\exp\left(-\frac{\psi(u_i-1)^2}{2u_i}\right)du_i$$
$$=\left(\prod_{j=1}^{m_i-1}\frac{\Lambda_{ij}^{d_{ij}}}{d_{ij}!}\right)\int_0^\infty u_i^{d_i}\left(\frac{\psi}{2\pi u_i^3}\right)^{\frac{1}{2}}\exp\left(-\frac{\psi}{2u_i}\left((u_i-1)^2+\frac{2u_i^2\Lambda_i}{\psi}\right)\right)du_i$$
$$=\left(\prod_{j=0}^{m_i-1}\frac{\Lambda_{ij}^{d_{ij}}}{d_{ij}!}\right)\int_0^\infty u_i^{d_i}\left(\frac{\psi}{2\pi u_i^3}\right)^{\frac{1}{2}}\exp\left(-\frac{\psi}{2u_i}\left(\left(1+\frac{2\Lambda_i}{\psi}\right)u_i^2-2u_i\sqrt{1+\frac{2\Lambda_i}{\psi}}+1\right)+\psi-\psi\sqrt{1+\frac{2\Lambda_i}{\psi}}\right)du_i$$
$$=\left(\prod_{j=0}^{m_i-1}\frac{\Lambda_{ij}^{d_{ij}}}{d_{ij}!}\right)\int_0^\infty u_i^{d_i}\left(\frac{\psi}{2\pi u_i^3}\right)^{\frac{1}{2}}\exp\left(\psi\left(1-\sqrt{1+\frac{2\Lambda_i}{\psi}}\right)\right)\exp\left(-\frac{\psi}{2u_i}\left(u_i\sqrt{1+\frac{2\Lambda_i}{\psi}}-1\right)^2\right)$$
$$=\left(\prod_{j=0}^{m_i-1}\frac{\Lambda_{ij}^{d_{ij}}}{d_{ij}!}\right)\exp\left(\psi\left(1-\sqrt{1+\frac{2\Lambda_i}{\psi}}\right)\right)m\left(d_i,\left(1+\frac{2\Lambda_i}{\psi}\right)^{-\frac{1}{2}},\psi\right)$$

where $m\left(d_i,\left(1+\frac{2\Lambda_i}{\psi}\right)^{-\frac{1}{2}},\psi\right)$ denotes the d_ith moment of an IG distribution with mean $\left(1+\frac{2\Lambda_i}{\psi}\right)^{-\frac{1}{2}}$ and variance $\frac{1}{\psi}\left(1+\frac{2\Lambda_i}{\psi}\right)^{-\frac{3}{2}}$. The nth raw moment of an IG distribution given by Wolfram (2005) is

$$m(n,\mu,\psi)=\exp\left(\frac{\psi}{\mu}\right)\sqrt{\frac{2\psi}{\pi}}\mu^{n-\frac{1}{2}}K_{\frac{1}{2}-n}\left(\frac{\psi}{\mu}\right)$$

where $K_n(z)$ is a modified Bessel function of the second kind. It then follows that the integral expression is equivalent to

$$\left(\prod_{j=0}^{m_i-1}\frac{\Lambda_{ij}^{d_{ij}}}{d_{ij}!}\right)\exp(\psi)\sqrt{\frac{2\psi}{\pi}}\left(1+\frac{2\Lambda_i}{\psi}\right)^{-\frac{1}{2}(d_i-\frac{1}{2})}K_{\frac{1}{2}-d_i}\left(\psi\sqrt{1+\frac{2\Lambda_i}{\psi}}\right).$$

A more detailed discussion of the estimating equations can be found in Shoukri *et al.* (2004). The modified Bessel function of the second kind for half integers can also be expressed as a finite sum,

$$K_{\frac{1}{2}-n}(z)=\sqrt{\frac{\pi}{2z}}\exp(-z)\sum_{j=0}^{n-1}(2z)^{-j}\frac{\Gamma(n+j)}{\Gamma(n-j)j!}$$

see Erdelyi (1953) for more details. When $d_i=0$ the integral expression evaluates to

$$\exp\left(\psi\left(1-\sqrt{1+\frac{2\Lambda_i}{\psi}}\right)\right)$$

which is seen as $m\left(0,\left(1+\frac{2\Lambda_i}{\psi}\right)^{-\frac{1}{2}},\psi\right)=1$ in this case.

Continuous part of the CP

$$\left(\prod_{j=1}^{m_i-1}\frac{\Lambda_{ij}^{d_{ij}}}{d_{ij}!}\right)\int_0^\infty u_i^{d_i-1}\exp\left(-\Lambda_i u_i\right)\exp(-\rho-\nu u_i)\sum_{j=1}^\infty\frac{\rho^j(\nu u_i)^j}{\Gamma(j)j!}\,du_i$$
$$=\left(\prod_{j=1}^{m_i-1}\frac{\Lambda_{ij}^{d_{ij}}}{d_{ij}!}\right)\int_0^\infty \exp(-\rho-(\nu+\Lambda_i)u_i)\sum_{j=1}^\infty\frac{(\rho\nu)^j u_i^{j+d_i-1}}{\Gamma(j)j!}\,du_i$$
$$=\left(\prod_{j=1}^{m_i-1}\frac{\Lambda_{ij}^{d_{ij}}}{d_{ij}!}\right)\exp(-\rho)\sum_{j=1}^\infty\frac{(\rho\nu)^j}{\Gamma(j)j!}\int_0^\infty u_i^{j+d_i-1}\exp(-(\nu+\Lambda_i)u_i)\,du_i$$
$$=\left(\prod_{j=1}^{m_i-1}\frac{\Lambda_{ij}^{d_{ij}}}{d_{ij}!}\right)\exp(-\rho)\sum_{j=1}^\infty\frac{(\rho\nu)^j}{\Gamma(j)j!}(\nu+\lambda_i)^{-(d_i+j)}(d_i+j-1)!$$
$$=\left(\prod_{j=1}^{m_i-1}\frac{\lambda_{ij}^{d_{ij}}}{d_{ij}!}\right)\frac{\exp(-\rho)}{(\nu+\Lambda_i)^{d_i}}\sum_{j=1}^\infty\frac{(d_i+j-1)!}{\Gamma(j)j!}\left(\frac{\rho\nu}{\nu+\Lambda_i}\right)^j.$$

To evaluate this expression, consider for $x\neq 0$

$$\exp\left(\frac{1}{x}\right)=\sum_{j=0}^\infty\frac{1}{j!x^j}.$$

By then taking the nth derivative of both sides,

$$\frac{d^n}{dx^n}\exp\left(\frac{1}{x}\right)=\frac{(-1)^n}{x^n}\sum_{j=1}^\infty\frac{(n+j-1)!}{(j-1)!j!x^j}$$

the following equivalence results

$$\frac{\exp(-\rho)}{(\nu+\Lambda_i)^{d_i}}\sum_{j=1}^\infty\frac{(d_i+j-1)!}{\Gamma(j)j!}\left(\frac{\rho\nu}{\nu+\Lambda_i}\right)^j=\frac{\exp(-\rho)}{(-\rho\nu)^{d_i}}\left[\frac{d^{d_i}}{dx^{d_i}}\exp\left(\frac{1}{x}\right)\right]_{x=\frac{\nu+\Lambda_i}{\rho\nu}}.$$

Finally, it is shown via induction that

$$\frac{d^n}{dx^n}\exp\left(\frac{1}{x}\right)=(-1)^n\exp\left(\frac{1}{x}\right)\sum_{j=0}^{n-1}j!\binom{n}{j}\binom{n-1}{j}x^{j-2n}$$

$$\frac{d^{n+1}}{dx^{n+1}}\exp\left(\frac{1}{x}\right)=\frac{d}{dx}(-1)^n\exp\left(\frac{1}{x}\right)\sum_{j=0}^{n-1}j!\binom{n}{j}\binom{n-1}{j}x^{j-2n}$$
$$=(-1)^{n+1}\exp\left(\frac{1}{x}\right)\sum_{j=0}^{n-1}j!\binom{n}{j}\binom{n-1}{j}x^{-2(n+1)+j}$$
$$+(-1)^{n+1}\exp\left(\frac{1}{x}\right)\sum_{j=0}^{n-1}j!\binom{n}{j}\binom{n-1}{j}(2n-j)x^{j-2n-1}$$
$$=(-1)^{n+1}\exp\left(\frac{1}{x}\right)\sum_{j=0}^{n-1}j!\binom{n}{j}\binom{n-1}{j}x^{-2(n+1)+j}$$
$$+(-1)^{n+1}\exp\left(\frac{1}{x}\right)\sum_{k=1}^{n}(k-1)!\binom{n}{k-1}\binom{n-1}{k-1}(2n-k+1)x^{-2(n+1)+k}$$

where the last line follows from the change of variables $j = k - 1$. Now consider the coefficient of $x^{-2(n+1)+l}$, that is

$$
\begin{aligned}
& l!\binom{n}{l}\binom{n-1}{l} + (l-1)!\binom{n}{l-1}\binom{n-1}{l-1}(2n-l+1) \\
=& l!\binom{n}{l}\left(\binom{n-1}{l} + \binom{n-1}{l-1}\frac{2n-l+1}{n-l+1}\right) \\
=& l!\binom{n}{l}\left(\frac{(n-1)!n(n+1)}{l!(n+l-1)!}\right) \\
=& l!\binom{n}{l}\binom{n+1}{l}.
\end{aligned}
$$

Therefore the integral expression for $d_i > 0$ is equivalent to

$$
\left(\prod_{j=1}^{m_i-1}\frac{\Lambda_{ij}^{d_{ij}}}{d_{ij}!}\right)\frac{1}{(\rho\nu)^{d_i}}\exp\left(-\rho\left(1-\frac{\nu}{\nu+\Lambda_i}\right)\right)\sum_{j=0}^{d_i-1} j!\binom{d_i}{j}\binom{d_i-1}{j}\left(\frac{\nu+\Lambda_i}{\rho\nu}\right)^{j-2d_i}.
$$

If $d_i = 0$, the integral expression is equivalent to

$$
\exp(-\rho)\left(\exp\left(\frac{\rho\nu}{\nu+\Lambda_i}\right)-1\right).
$$

Web Appendix B

The Laplace transform of the compound Poisson distribution described in Section 3.1 is

$$
\mathbb{E}\left(\exp(-sU_i)\right) = \exp\left(-\frac{\rho s}{\nu+s}\right).
$$

This can be seen by replacing $m = 1$ in the Laplace transform of the power variance family function also described in Section 3.1. For any finite $\nu > 0$ and s such that $\frac{s}{\nu+s} > 0$, it is clear that the Laplace transform of the compound Poisson distribution will tend to 0 as $\rho \to \infty$ regardless of the value of ν. Hence ν becomes irrelevant under the null hypothesis $H_0 : \rho = \infty$.
